# Outpatient medications associated with protection from COVID-19 hospitalization

**DOI:** 10.1371/journal.pone.0282961

**Published:** 2023-03-31

**Authors:** Harpal Singh Sandhu, Joshua Lambert, Zach Steckler, Lee Park, Arnold Stromberg, Julio Ramirez, Chi-fu Jeffrey Yang

**Affiliations:** 1 Department of Bioengineering, University of Louisville Speed School of Engineering, Louisville, KY, United States of America; 2 University of Cincinnati College of Nursing, Cincinnati, OH, United States of America; 3 Dr. Bing Zhang Department of Statistics, University of Kentucky, Lexington, KY, United States of America; 4 Norton Infectious Diseases Institute, Norton Hospital, Louisville, KY, United States of America; 5 Department of Surgery, Harvard Medical School, Boston, MA, United States of America; Pikeville Medical Center, UNITED STATES

## Abstract

The COVID-19 pandemic remains the pre-eminent global health problem, and yet after more than three years there is still no prophylactic agent against the disease aside from vaccines. The objective of this study was to evaluate whether pre-existing, outpatient medications approved by the US Food and Drug Administration (FDA) reduce the risk of hospitalization due to COVID-19. This was a retrospective cohort study of patients from across the United States infected with COVID-19 in the year 2020. The main outcome was adjusted odds of hospitalization for COVID-19 amongst those positive for the infection. Outcomes were adjusted for known risk factors for severe disease. 3,974,272 patients aged 18 or older with a diagnosis of COVID-19 in 2020 met our inclusion criteria and were included in the analysis. Mean age was 50.7 (SD 18). Of this group, 290,348 patients (7.3%) were hospitalized due to COVID-19, similar to the CDC’s reported estimate (7.5%). Four drugs showed protective effects against COVID-19 hospitalization: rosuvastatin (aOR 0.91, p = 0.00000024), empagliflozin-metformin (aOR 0.69, p = 0.003), metformin (aOR 0.97, p = 0.017), and enoxaparin (aOR 0.88, p = 0.0048). Several pre-existing medications for outpatient use may reduce severity of disease and protect against COVID-19 hospitalization. Well-designed clinical trials are needed to assess the efficacy of these agents in a therapeutic or prophylactic setting.

## Introduction

Coronavirus disease 2019 (COVID-19) represents a true global health emergency without precedent in living memory, and yet almost two years into the pandemic there are still few antiviral therapies against the disease or for pre- or post-exposure prophylaxis. Repurposing already existing drugs to these ends is an alternative to *de novo* drug discovery that could have a significant impact on the course of the pandemic. *In vitro*, *in vivo*, *in silico* studies, and theoretical considerations have shown that there are several hundred US Food and Drug Administration (FDA)-approved drugs that have an antiviral effect on severe acute respiratory syndrome coronavirus 2 (SARS-CoV-2), the causative virus of COVID-19, or the pathophysiology it causes.

Numerous studies have utilized computer simulations to model the biochemical interaction of SARS-CoV-2 and cellular proteins with known small-molecule drugs, a so-called *in silico* approach [1–10]. These studies rapidly generated a list of promising antiviral chemical entities, but they provided no *in vitro*, *in vivo*, or human clinical data for drug efficacy in COVID-19. Moreover, a strong chemical interaction between a drug and SARS-CoV-2 is no guarantee of any therapeutic effect. Other *in silico* studies have expanded the list of promising targets by examining the “interactome” of SARS-CoV-2 [11, 12], which represents the large web of receptors, signaling molecules, and proteins involved in the pathobiology of the virus. Since molecules downstream in this process may be druggable targets, this approach logically expands the range of possible therapeutics. Unfortunately, it suffers from similar flaws as *in silico* studies, namely a lack of *in vivo* or clinical data. High-throughput screening of drugs to prevent viral infection *in vitro* has identified some molecules as more promising antivirals than others, including drugs previously used for treating other epidemic coronavirus infections [13–26]. Some of these studies were the basis for clinical trials repurposing hydroxychloroquine, lopinavir-ritonavir, azithromycin, type I interferons, and remdesivir against COVID-19 [27–32]. All of these have failed to show any efficacy except remdesivir, which hastened recovery in one clinical trial but sadly provides no mortality benefit [33].

Finally, these initial *in silico* and *in vitro* approaches are limited to predictions of 1^st^ and 2^nd^ degree antiviral effects. None of the predictions can identify drugs that may have pleotropic benefits in preventing complications of COVID-19 disease or organ-specific pathology. However, it is now well-recognized that severe COVID-19 pathology extends well beyond the respiratory epithelium targeted by SARS-CoV-2 infection. The hyperinflammatory cytokine release syndrome (CRS) induced by COVID-19 is a major contributor to respiratory and multiorgan failure [34–39]. Furthermore, COVID-19-associated coagulopathy and vasculopathy are also major causes of cerebral, cardiovascular, renal, and other end-organ damage [40–45].

While clinical trials are ongoing, they proceed slowly, require intensive resources, and can only test a limited number of these potential therapies at any given time. Hence, there is an urgent need for clinical data on the effects of this wide range of potentially efficacious drugs. A big data approach allows us to rapidly mine retrospective data to assess drug effects with clinical data. With this goal in mind, we sought to determine if there are pre-existing, outpatient medications approved by the FDA that reduce the odds of hospitalization due to COVID-19.

## Methods

### Data source

The data for this study were derived from a large health insurance claims database, Symphony Health, provided by the COVID-19 Research Database [46]. Data from late 2018 through mid-2021 from the United States were included. While of course COVID-19 did not exist in 2018, data prior to 2018 was still used to gather important clinical data on these patients, specifically what medical diagnoses they carried and thus what comorbidities they may or may not have had. No Medicaid or Medicare data were available in the Symphony Health dataset. For a given patient, the dataset contains all his/her international classification of diseases 10 (ICD10) diagnosis codes entered by any healthcare provider for the patient, all current procedural terminology (CPT) codes, records of all hospitalizations, and all outpatient and inpatient prescription drug data. Thus, essentially all drug data, hospitalizations, past medical history, active medical issues, procedures (e.g. intubation), and surgeries are captured for all patients within the dataset.

### Study population

All patients aged 18 or older with the ICD10 code for COVID-19 (U07.1) in 2020 within the Symphony Healthcare database were included in the analysis. All cases from January 1, 2021, and onwards were excluded because of the roll out of the COVID-19 mass vaccination campaign in the United States. Not only does the adaptive immunity conferred by vaccination decrease the number of hospitalized cases and thus effectively decrease our ability to detect drug signals, but also this immunity, in theory, introduces a further interaction variable with the drugs themselves, a complicating factor we wished to avoid during a broad study of main drug effects.

This was a purely retrospective study using de-identified data from a third party. As a result, this study was deemed exempt from review by the institutional review board of the University of Louisville. Similarly, the need for informed consent was waived.

### Medications evaluated

A total of 159 different outpatient medications with potential therapeutic effects against COVID-19, based on *in vitro*, *in vivo*, *in silico* data, or theoretical considerations, were studied. These drugs fell into seven broad categories: antimicrobials, immunomodulatory agents, combined antimicrobial/immunomodulatory agents, anti-cancer drugs, anticoagulants and antiplatelet drugs, anti-androgen agents, and a miscellaneous group (Table 1). A full list of these drugs can be found in the supplemental materials. All patients had received a prescription for the medication within at least 90 days prior to being diagnosed with COVID-19.

**Table 1:** Number of drugs studied by class.

Drug Class	Number
Antimicrobials	35
Immunomodulatory (IMT) agents	20
Combined IMT and antimicrobial agents	19
Anti-cancer drugs	23
Anticoagulants and antiplatelet agents	14
Anti-androgens	14
Miscellaneous	34

The 159 drugs studied broadly fell into six different drug classes plus a seventh miscellaneous group. Number of drugs studied within each class are listed.

### Primary outcome

The main outcome was hospitalization due to COVID-19. A hospitalized case of COVID-19 was defined using a claims-specific event reconstruction strategy which utilized the ICD10 and CPT codes available from Symphony health. From the claims data, we categorized a patient as hospitalized due to COVID-19 if the patient had a hospitalization CPT code (99221, 99222, or 99223) up to 14 days *after* a COVID diagnosis (U07.1) and at least one code for acute respiratory illnesses. We also included cases where a COVID diagnosis code was entered up to two days into hospitalization, as many cases are not diagnosed until the patient has presented to the hospital. The additional diagnoses required for inclusion were one of pneumonia due to SARS-associated coronavirus (J12.81), other viral pneumonia (J12.89), acute bronchitis due to other specified organism (J20.8), bronchitis not specified as acute or chronic (J40), unspecified acute lower respiratory infection (J22), other specified respiratory disorders (J98.9), or acute respiratory distress syndrome (J80). Notably, claims data do not include laboratory results, and thus we rely on a treating physician’s correct use of the above codes to identify cases. A comprehensive overview of this case definition and the strategy that precedes it can be found elsewhere [47].

### Statistical analysis

Multivariable logistic regression was performed adjusting for known risk factors for more severe disease due to COVID-19: age, biological sex, hypertension, diabetes mellitus (DM), obesity, coronary artery disease and congestive heart failure, stroke, chronic pulmonary, liver, kidney disease, organ transplantation, stroke, and certain cancers. The first digit of USPS zip code, a surrogate for geographic region, was also a co-variate for which we adjusted, because regional variation in public health policy is another potential variable in outcomes. Statistical testing to adjust for multiple comparisons was made using the false discovery rate. All statistics were performed using the software R version 4.0.

It was not appropriate or practical to involve patients or the public in the design or conduct of this study as it was a retrospective cohort study using data from a third-party database.

## Results

**Fig 1:**
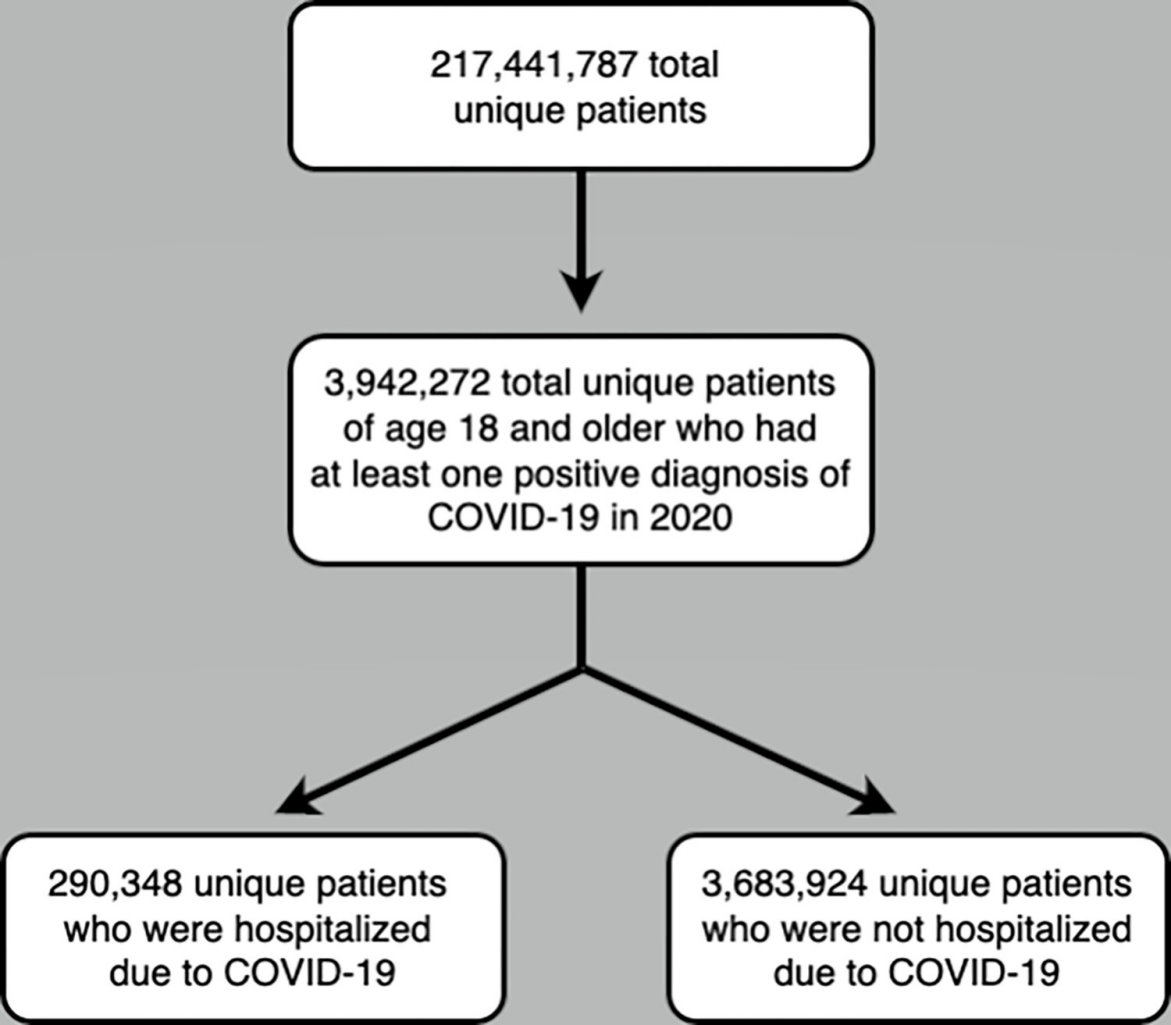
Flow chart showing the total number of patients in the database and exclusions that result in the final number of patients included in the study.

The Symphony database contains data on 217,441,787 unique Americans. Of these, 3,974,272 adult patients carried a diagnosis code for COVID-19 in 2020 and were included in the analysis (Fig 1). Mean age of the COVID-positive cohort was 50.7 years (SD 18), and 55% were female (Table 2). Hypertension (38.7%), diabetes mellitus (22.5%), obesity (12.2%), cardiovascular disease (11.1%), and pulmonary disease (10.6%) were the most common comorbidities (Table 3). Inclusion criteria for patients were a positive COVID-19 diagnosis between January 1^st^, 2020, and December 31^st^, 2020, and age of at least 18 in 2020. Of these COVID-19-positive patients, 290,384 were hospitalized due to COVID-19 (7.3%) and 3,683,924 were not hospitalized due to COVID-19 (Table 2). Average age of the sample was 50.72 years, with a small majority (55.2%) of the sample being female. The patients hospitalized due to COVID-19 tended to be older (mean age of 63.57 years compared to 49.71 years for those not hospitalized), and noticeably greater proportions of patients hospitalized had pre-existing health conditions or an organ transplant compared to those patients not hospitalized.

**Table 2:** Baseline demographics overall, hospitalized due to COVID-19, and not hospitalized due to COVID-19.

		COVID-19 Patients		Hospitalized Due to COVID-19		Not Hospitalized Due to COVID-19	
		N	Percentage (%)	N	Percentage (%)	N	Percentage (%)
Total		3,974,272	100.0	290,348	7.31	3,683,924	92.69
**By age group**							
	*18–49*	1,867,749	47.0	49,638	17.10	1,818,111	49.35
	*50–64*	1,045,133	26.3	85,271	29.37	959,862	26.06
	*65–80*	1,061,390	26.7	155,439	53.54	905,951	24.59
	*Average age*	50.72		63.57		49.71	
**By gender**							
	*Male*	1,781,125	44.8	132,298	45.57	1,623,076	44.06
	*Female*	2,193,114	55.2	158,049	54.43	2,060,816	55.94
**By pre-existing condition/procedure**							
	*Hypertension*	1,539,797	38.7	205095	70.64	1334702	36.23
	*Diabetes*	895,152	22.5	139836	48.16	755316	20.50
	*Obesity*	485,005	12.2	71256	24.54	413749	11.23
	*Cardiovascular*	441,541	11.1	75668	26.06	365873	9.93
	*Pulmonary disease*	421,478	10.6	56649	19.51	364829	9.90
	*Renal*	275,512	6.9	54096	18.63	221416	6.01
	*Stroke*	78,256	2.0	12738	4.39	65518	1.78
	*Cancer*	40,342	1.0	7891	2.72	32451	0.88
	*Liver*	26,735	0.7	4410	1.52	22325	0.61
	*Organ transplant*	13,505	0.3	2932	1.01	10573	0.29

**Table 3:** Drugs found to be statistically significant (p < 0.05) in preventing hospitalization due to COVID-19: Number of patients on drug, adjusted odds ratios, p-values, and Bonferroni 95% confidence intervals for drugs. Statistically significant results (p < 0.05) are bolded.

Drugs	Number of patients on drug	Adjusted Odd ratios (95% CI)	p-value	Multiple-testing correction p-values	
				False Discovery Rate (FDR) Correction	Bonferroni Correction
Rosuvastatin	35,322	0.907 (0.876–0.938)	**< 0.0001**	**< 0.0001**	**< 0.0001**
Empagliflozin- Metformin	1,090	0.691 (0.559–0.855)	**0.0007**	**0.0032**	0.0959
Enoxaparin	7,907	0.884 (0.822–0.952)	**0.0011**	**0.0048**	0.1544
Metformin	102,288	0.972 (0.953–0.991)	**0.0042**	**0.0169**	0.6089

The normal-based and false discovery rate p-values (Table 3) suggest that four drugs are statistically significant (p < 0.05) in our analysis, in reducing risk of hospitalization due to COVID-19: Rosuvastatin, empagliflozin-metformin, enoxaparin, and metformin. When the Bonferroni p-value is considered, only one drug was statistically significant (p < 0.05): rosuvastatin. The adjusted odds ratio estimate for rosuvastatin was 0.91.

Metformin was the most common drug of the four protective agents with 102,288 patients on the medication, but all four had numbers over 1000. No other statin showed protective effects, and statins as a class were not protective.

Four drugs showed protective effects against COVID-19 hospitalization after adjusting for covariates and for multiple comparisons with the false discovery rate (Table 3). The statin rosuvastatin showed an adjusted odds ratio (aOR) of 0.91, the combination hypoglycemic empagliflozin-metformin 0.69, metformin alone 0.97, and the low-molecular weight heparin enoxaparin 0.88. Empagliflozin alone was not associated with a lower risk of hospitalization. When statistically adjusting for multiple comparisons with the Bonferroni correction, only rosuvastatin was significant.

## Discussion

In this large, population-based study of outpatient drugs effects in COVID-19, we found that four of the 159 drugs with plausibly beneficial effects conferred protection against hospitalization for COVID-19 after adjusting for multiple risk factors for COVID-19 and for multiple comparisons. These include the statin rosuvastatin, the classic hypoglycemic metformin, whose effect was greater when combined with empagliflozin, and the anticoagulant enoxaparin.

Each of these drugs has its own biologically plausible mechanism by which it may protect against severe COVID-19. While statins are typically prescribed to lower lipid levels, they have a broad range of immunomodulatory, anti-inflammatory, and anti-thrombotic effects. Endotheliopathy is a major feature of COVID-19. High levels of multiple circulating cytokines activate endothelial cells (ECs), as does recognition of viral RNA by toll-like receptors on ECs. Actual viral infection of ECs also directly damages them, leading to a local endotheliitis in some organ systems and increasing the risk of thrombotic events [48]. A recent nationwide retrospective study of outpatient statin users in France, of whom there were over 2 million, similarly showed that the risk of hospitalization after adjustment was 16% lower in statin users than non-users with the effect size largest for fluvastatin, showing a 25% adjusted HR reduction [49]. Almost 40 trials of statins in COVID-19 are underway, but very few have reported final results yet [50]. Two placebo-controlled trials of atorvastatin 20–40 mg in hospitalized COVID-19 patients found no beneficial effect [51, 52]. More relevant to this work, none is a prophylactic study.

The prominence of neutrophil extracellular traps (NETs) in COVID pathophysiology is another contributor to thrombosis [53]. These features of disease drive the high rates of pulmonary emboli, stroke, and other vascular occlusions in the disease. Any protective effect of enoxaparin is likely mediated through its direct anticoagulant effect. Rosuvastatin may similarly be exerting an antithrombotic effect, but its other properties suggest it could be acting via multiple pathways. The drug also downregulates inflammation driven by the nuclear factor kappa B (NF-κB) transcription factor, a pathway that is strongly upregulated in COVID [54, 55], and it has stabilizing effects on the endothelium [56]. Earlier in the pandemic enoxaparin had been assessed at prophylactic and therapeutic doses in multiple randomized controlled trials (RCT) in the inpatient setting with mixed results [57, 58], and several trials are still ongoing. At least one trial of colchicine plus rosuvastatin for inpatients is underway [59]. At this point, use of prophylactic doses of anticoagulation has become routine in patients hospitalized for COVID-19 [60]. While enoxaparin’s protective effect herein strengthens the connection between thrombotic processes and morbidity in COVID-19 pathophysiology, it is hard to argue that even low-dose anticoagulation would have a preventive role prior to COVID exposure and infection due to the many risks of this class of therapy.

Metformin is a biguanide that is familiar to all physicians, a staple of type II DM therapy. While its ability to inhibit hepatic gluconeogenesis and improve insulin sensitivity are well known, its beneficial effects for other diseases are legion. Preclinical and observational data strongly suggest that metformin has anti-neoplastic, antimicrobial, and anti-senescent properties [61–63]. The drug alters the conformation of the ACE2 receptor via AMP kinase, which potentially inhibits SARS-CoV-2 entry into cells, decreases viral replication by increasing endosomal pH, decreases systemic inflammation via reductions in NETosis and serum IL-6 and TNF-α levels, decreases thrombosis by inhibiting platelet activation factor and through the aforementioned effect on NETs, and resolves lung inflammation with less pulmonary fibrosis through its effect on local expression of TGF-β and vascular endothelial growth factor [64]. Which of these many pleiotropic effects is dominant, how they interact with one another, and in what sorts of clinical scenarios they are most helpful remain unknown. A recently completed, large factorial trial of metformin, ivermectin, and fluvoxamine in outpatients infected with COVID-19 showed a trend towards benefit with metformin, and in particular demonstrated a 42% reduction in risk of emergency department visit, hospitalization, or death [65].

We emphasize that our results suggest drugs that may have effects as preventative or mitigating agents, as all patients in this study were prescribed these drugs *prior* to a diagnosis of COVID-19. This stands in contrast to therapeutic agents started after symptomatic disease develops, which is the context of most clinical trials. As such, these results generate hypotheses and suggest other drugs for prospective study.

Strengths of this study include its very large sample size, the large number of drugs studied, *a prior* basis for interest in each drug, the rigorous statistical adjustments for multiple comparisons, and adjustment for established risk factors for severe COVID-19. To the best of our knowledge, this is the largest pharmacoepidemiological study of COVID-19 performed thus far. Its principal weakness is that it suffers from the risk of residual confounding inherent to all retrospective cohort studies. Furthermore, claims-based data are imperfect, and some small but unknown fraction of cases are miscoded, thereby diluting drug effects. There may also be a bias towards more severe disease in this study. Some COVID-positive patients with milder disease are diagnosed in a variety of testing settings in the community and may never present to a healthcare provider for COVID-related care. As a result, healthcare claims with a COVID-19 ICD10 code might never be recorded for these patients. Moreover, the US hospital system was severely strained in different regions at different times during 2020. It is possible that less severe cases of COVID-19 that presented to some US emergency departments at various times in 2020 may have been discharged for outpatient follow up when under more normal circumstances they might have been admitted. This could affect our main outcome measure, hospitalization. Since most hospitals in the US were not facing excessive patient volume during 2020, we expect this effect to be small. To the extent it does exist, it would raise the threshold for hospitalization and suggest that the four drugs that were significant herein have effects on particularly severe disease.

Drug dosage is a further issue in the study. The dataset shows prescriptions dispensed to patients, but exact dosing is not known, only the dose of the tablet or formulation dispensed. For instance, a patient dispensed metformin in 500 mg tablets may be taking 500 mg PO BID or 1000 mg PO BID, and thus dose-response relationships cannot be assessed. While almost all the agents studied are chronic medications, and thus one can reasonably assume that the drug has a biological effect at the time of infection, the same is not true of antimicrobials. Most antimicrobial use is for short, defined periods of time, and thus one is left with a degree of uncertainty regarding what level of biological effect these antecedent prescriptions of antimicrobials may have had at the time of infection.

We did not perform any subgroup analyses within this study. It is possible than some of these agents are more potent in patients of a certain age or sex, or those with particular co-morbidities. It is also possible that some of the drugs that were not significant may show very strong effects in certain subgroups, but that the significance of any such effects were diluted out by the general population. We are in the process of conducting multiple subgroup analyses on a second dataset to answer precisely these questions.

While we do not consider generalizability of this study’s findings to the wider US population to be a weakness per se, one should recognize the features of this database originating from private health insurers. Private health insurance in the US does cover the majority of the US population, but in general this is a slightly healthier cohort of higher socioeconomic status than those enrolled in public insurance programs and the uninsured. In this cohort, 7.3% of patients with COVID-19 were hospitalized, very similar to the CDC’s reported estimate of 7.5% in 2020 [66]. We believe this validates our methods and dataset to a certain extent, and suggests some degree of generalizability. Last, analysis was deliberately limited to 2020 to avoid the complicating factor of vaccine-drug interactions, but that restriction also makes this work most applicable to the wildtype virus. Extrapolating these results to newer variants must be done with caution.

In summation, several pre-existing, FDA-approved medications for outpatient use may have protective effects against COVID-19. Well-designed clinical trials are needed to assess the efficacy of these agents in clinical practice.

## Supporting information

S1 Appendix A full list of all drugs evaluated in this study is included. (DOCX)
