# Supplementary material for: Outpatient medications associated with protection from COVID-19 hospitalization
Source: PLoS One. 2023 Mar 31;18(3):e0282961. doi: 10.1371/journal.pone.0282961 (PMC10065249; doi:10.1371/journal.pone.0282961)
Supplement: S1 Appendix — (DOCX) [file pone.0282961.s001.docx]

**List of Drugs Included for Study**

Antimicrobials

| mefloquine |
| --- |
| thiabendazole |
| nitazoxanide |
| bedaquiline |
| clofazamine |
| itraconazole |
| glecaprevir |
| grazoprevir |
| paritaprevir |
| simeprevir |
| sofosbuvir |
| dasabuvir |
| ribavirin |
| tenofovir |
| abacavir |
| emtricitabine |
| lamivudine |
| telbivudine |
| lopinavir |
| ritonavir |
| cobicistat |
| darunavir |
| atazanavir |
| raltegravir |
| paritaprevir |
| bictegravir |
| dolutegravir |
| efavirenz |
| nevirapine |
| rilpivirine |
| etravirine |
| doravirine |
| doxycycline |
| clarithromycin |
| trimethoprim-sulfamethoxazole |

Anti-cancer drugs

| dactinomycin |
| --- |
| imatinib |
| dasatinib |
| nilotinib |
| sunitinib |
| erlotinib |
| gemcitabine |
| decitabine |
| tamoxifen |
| toremifene |
| carflizomib |
| didanosine |
| temozolomide |
| thalidomide |
| lenalidomide |
| melphalan |
| ibrutinib |
| acalabrutinib |
| zanubrutinib |
| rilzabrutinib |
| duvelisib |
| selinexor |
| isotretinoin |
|  |

Immunomodulatory drugs

| tocilizumab |
| --- |
| sarilumab |
| anakinra |
| canakinumab |
| rilonacept |
| adalimumab |
| infliximab |
| golimumab |
| certolizumab |
| etanercept |
| abetacept |
| tofacitinib |
| ruxolitinib |
| fedratinib |
| upadacitinib |
| ocrelizumab |
| rituximab |
| ravulizumab |
| eculizumab |
| asunercept |
| fingolimod |

Drugs with combined immunomodulatory and antiviral effects

| azithromycin |
| --- |
| mycophenolic acid |
| hydroxychloroquine |
| chloroquine |
| sirolimus |
| tacrolimus |
| colchicine |
| leflunomide |
| mercaptopurine |
| mesalazine |
| methotrexate |
| baricitinib |
| celecoxib |
| valdecoxib |
| rofecoxib |
| indomethacin |
| methisazone |
| interferon-alpha |
| interferon-beta |
| ambrisentan |
| bosentan |
| macicentan |

Outpatient anticoagulants

| warfarin |
| --- |
| dabigatran |
| rivaroxaban |
| apixaban |
| edoxaban |
| betrixaban |
| fondaparinux |
| enoxaparin |

Antiplatelet agents

| dipyridamole |
| --- |
| aspirin |
| clopidogrel |
| ticagrelor |
| ticlopidine |
| prasugrel |

Anti-androgen agents

| degarelix |
| --- |
| leuprolide |
| goserelin |
| triptorelin |
| histrelin |
| abiraterone |
| ketoconazole |
| flutamide |
| bicalutamide |
| nilutamide |
| enzalutamide |
| apalutamide |
| darolutamide |
| dutasteride |

Miscellaneous

| ciclesonide |
| --- |
| fluticasone |
| vismodegib |
| camphor |
| equilin |
| paroxetine |
| carvedilol |
| quinacrine |
| eplerenone |
| oxymetholone |
| emodin |
| theophyline |
| famotidine |
| chlorpromazine |
| fluphenazine |
| promethazine |
| valproic acid |
| entacapone |
| verapamil |
| atorvastatin |
| simvastatin |
| rosuvastatin |
| pravastatin |
| lovastatin |
| degarelix |
| deferoxamine |
| tramadol |
| pamrevlumab |
| disulfiram |
| crizanlizumab |
| pentoxyphylline |
| linagliptine |
| dapagliflozin |
| metformin |
